# Update on the management of colchicine resistant Familial Mediterranean Fever (FMF)

**DOI:** 10.1186/s13023-019-1201-7

**Published:** 2019-10-15

**Authors:** Georges El Hasbani, Ali Jawad, Imad Uthman

**Affiliations:** 1 Division of Rheumatology, Department of Internal Medicine, American University of Beirut Medical Center, Beirut, Lebanon; 2 Department of Rheumatology, The Royal London Hospital, Bancroft Road, London, E1 4DG UK

**Keywords:** Familial Mediterranean fever, Autoinflammatory diseases, Colchicine resistance, Biologics

## Abstract

**Background:**

Familial Mediterranean Fever (FMF), an autoinflammatory disease, is characterized by self-limited inflammatory attacks of fever and polyserositis along with high acute phase response. Although colchicine remains the mainstay in treatment, intolerance and resistance in a certain portion of patients have been posing a problem for physicians.

**Main body:**

Like many autoimmune and autoinflammatory diseases, many colchicine-resistant or intolerant FMF cases have been successfully treated with biologics. In addition, many studies have tested the efficacy of biologics in treating FMF manifestations.

**Conclusion:**

Since carriers of FMF show significantly elevated levels of serum TNF alpha, IL-1, and IL-6, FMF patients who failed colchicine were successfully treated with anti IL-1, anti IL-6, or TNF inhibitors drugs. It is best to use colchicine in combination with biologics.

## Background

Familial Mediterranean fever (FMF) (OMIM #249100) is the most common autoinflammatory disease (AID) worldwide [1]. The condition was first described in 1945 as “benign paroxysmal peritonitis” [2]. The typical phenotype of FMF includes self-limited inflammatory attacks of fever and polyserositis, arthritis, and dermal manifestations along with high acute phase response [3]. Although it has been classically known to affect people in the Mediterranean region like the Arabs, Armenians, Turks, Greeks, Italians, Persians, and Jews, FMF is seen worldwide due to travel and immigration that happened mainly in the twentieth century [4]. Clinically, FMF is highly heterogenous depending on the sequence variants in MEFV gene which is located on the short (p) arm of chromosome 16 encoding for a pyrin protein [5, 6]. Colchicine has been the mainstay of treatment for FMF since 1972 [7]. However, the molecular and genetic advancements have introduced new targeted drugs that could be used as an add-on to colchicine in certain circumstances such as resistance, which is defined as having 1 or more attacks per month despite receiving the maximally tolerated dose for ≥3 months. The objective of this review is to describe the different treatment modalities that have been successfully used in the course of management of colchicine resistant FMF patients.

## Main text

### Clinical picture and pathogenesis of FMF

FMF is characterized by self-limiting episodes of fever associated with serositis, arthritis, and dermal manifestations which last 12–72 h. The interval between the episodes is variable [8]. FMF has prodromal symptoms that occur 1–2 days prior to the onset of symptoms. These include constitutional, neuropsychiatric or physical signs, appetite and taste alterations, and pain in the region where the flare will appear [9]. The fever of FMF is high grade (> 38 °C), and is typically recurrent. It tends to rise rapidly followed by a plateau and rapid decrease over 1 to 3 days [9]. The peritoneal inflammation causes an abdominal pain that is initially localized and becomes generalized to resolve in 12 to 48 h. Pleuritis or pericarditis can cause chest pain. Pleuritic pain is unilateral, and lasts 12 to 48 h [10]. Pericarditis lasts longer than pleuritic plain for up to 14 days [11]. Arthritis is a common symptom that accompanies FMF attacks. It is usually monoarticular typically involving large joints of the lower limbs (knees and ankles) and develops in childhood [12]. The dermatological manifestations of FMF include painful and warm erysipelas-like skin lesion occurring on the lower limb about 10–35 cm^2^ in size with sharp boundaries. In children, those lesions may be the presenting feature of FMF [13]. Proteinuria can be developed in FMF patients. Kidney biopsy is recommended whenever the urinary protein is more than 0.5 g/24 h [14]. Renal amyloidosis is the major complication of FMF which leads to end-stage renal disease. Some of the risk factors for the development of amyloidosis are: Male gender, arthritis, delay in diagnosis, M694 V homozygous genotype, and family history of amyloidosis [15, 16].

In 1997, FMF was found to be associated with the MEFV gene on chromosome 16 [5, 6, 17]. The MEFV gene encodes for the protein pyrin/marenostrin which is an immunoregulatory molecule made up of 781 amino acids which interacts with caspase-1 and other inflammasome components to regulate interleukin IL-1β production. Inflammasomes are mutiprotein complexes that play a major role in both innate and adaptive immune systems [18]. 85% of FMF cases in the Mediterranean basin have genetic mutations encoded from exon 10 and exon 2 [4]. There are 9 clearly pathogenic variants of FMF which are: M694 V, M694I, M680I, V726A, R761H, A744S, I692del, E167D, and T267I. Other variants of unknown significance include: E148Q, K695R, P369S, F479 L, and I591T [19]. M694 V is the most common mutation in the Eastern Mediterranean populations, although less common among Arabs [20]. Since M694 V is associated with a severe disease phenotype, patients homozygous for M694 V are considered at a high risk for early disease [21].

### Diagnosis

The diagnosis of FMF is based on the Tel-Hashomer clinical criteria. Livneh et al. [22] noted that the Tel-Hashomer criteria includes typical, incomplete, and supportive cases. The diagnostic criterion for Yalcinkaya-Ozen has a better sensitivity than other criteria for FMF in children [23]. FMF attacks are classified into typical or incomplete. Typical attacks are defined as recurrent (≥3 of the same type), febrile (rectal temperature of 38 °C or higher), and short (lasting between 12 h and 3 days). Incomplete attacks are defined as painful and recurrent attacks that differ from typical attacks in one or two features, as follows:

1. The temperature is normal or lower than 38 °C
2. The attacks are longer or shorter than specified (but not shorter than 6 h or longer than a week)
3. No signs of peritonitis during the abdominal attacks
4. The abdominal attacks are localized
5. The arthritis involves joints other than those specified

The attacks that do not fulfill the definition for a typical or incomplete attack are not considered as FMF attack [24]. Mediterranean fever genetic testing can be useful to detect at least two heterozygote mutations or a homozygous mutation.

### Management

In January 2016, The European League Against Rheumatism (EULAR) recommendation set for the management of FMF has been published supported by the best available evidence [25]. The goal of FMF treatment, as per the EULAR recommendations, is to obtain the control of acute attacks, minimize the chronic and subclinical inflammation, prevent complications, and provide an acceptable quality of life.

#### Colchicine: diagnostic and therapeutic limitations

Colchicine has been the main treatment of FMF since 1972 [26]. Colchicine is related to pyrin through altering the organization of actin cytoskeleton by binding to tubulin monomers and inhibiting polymer formation [27, 28]. Although colchicine cannot completely prevent febrile episodes, its use may halt the progression of amyloidosis, reversing proteinuria in the absence of irreversible glomerular damage [29]. Colchicine has a narrow therapeutic index. Sometimes, its maximum tolerated dose may not be adequate to control disease activity. Gastrointestinal disturbance may be seen in up to 10% of patients in the first month of treatment which might lead to increased fecal excretion of starch, fat, and bile acids and decreased absorption of D-xylose and vitamin B12 [30, 31]. Merlin et al. [32] case report suggested that colchicine is associated with azoospermia at high doses. However, men need not stop colchicine prior to conception [25]. In women, colchicine use is safe during pregnancy and lactation [33–35]. Nevertheless, it should be used cautiously in patients with impaired renal or hepatic functions [36]. Compliance with colchicine is very important for the proper management of FMF. Although colchicine is effective for FMF, approximately one-third of the patients treated with colchicine have a partial remission, and about 5–10% are non-responders; another 2–5% do not tolerate the drug mainly due to gastrointestinal symptoms [37]. Barut et al. recent study showed that the frequency of M694 V homozygosity might be associated with non-responding to colchicine [38]. Because FMF is the most common autoinflammatory disease, colchicine resistance or unresponsiveness posed a problem for physicians. Since carriers of FMF show significantly elevated levels of serum TNF, IL-1, IL-6 and IL-8, new biological drugs targeting those cytokines were used in colchicine non-responders or resistant [39].

Proper management of FMF includes trying colchicine up to 2 mg a day until the flare settles. The dose is reduced to 0.5 or 1 mg daily in the time when CRP or preferably serum AA protein is checked weekly for at least 8 weeks to see whether the acute-phase response is high. In that case, treatment is escalated with a higher dose of colchicine. If there is no control of FMF manifestations, other treatments are added to a low dose of colchicine [25]. Patients who continue to have ≥1 attacks per month despite receiving the maximally tolerated dose for ≥3 months might be considered non-responder or resistant to colchicine [25]. In those patients, biologics and maximal tolerated dose of colchicine are recommended [25]. In cases of AA amyloidosis secondary to FMF addition, the treatment also should be intensified with biologics and maximal tolerated dose of colchicine [25].

#### Anti Il-1 drugs

Because elevated levels of IL-1 are related to inflammatory activity, the use of drugs targeting IL-1 have been proposed. Three different types of IL-1 receptor antagonists are available. Anakinra is a human recombinant un-glycosylated analog of the IL-1 receptor antagonist. Rilonacept is a fusion protein that contains the extracellular portions of type I IL-1receptor and IL-1 receptor accessory protein. Canakinumab is a fully humanized monoclonal antibody of the class IgG1 that acts specifically against IL-1 beta [40].

##### Anakinra

Before 2003, Anakinra was successfully tested for the treatment of multiple autoimmune diseases including rheumatoid arthritis and systemic lupus erythematosus (SLE). In 2003, 5 patients with Muckle-Wells Syndrome which is a milder form of FMF responded successfully to a trial of Anakinra [41]. The first quantitative study discussing the efficacy of blocking IL-1 receptors in FMF came by Chae et al. [42]. Anakinra suppressed the acute-phase proteins in a patient with FMF and amyloidosis supporting a direct effect of the mutated protein in FMF pyrin on IL-1beta activation suggesting a heightened IL-1 responsiveness as one factor selecting for pyrin mutations. Shortly after that, the efficacy of Anakinra in the treatment of a colchicine resistant 68 year old woman homozygous for the M694 V mutation of the MEFV gene [43] and a 15 year-old colchicine resistant girl was reported [44]. Table 1 summarizes all case reports and studies describing the use of Anakinra in treating the manifestations of FMF.

**Table 1:** Studies and case reports that discussed the use of Anakinra in FMF

Study	Description	Follow-up
Chae et al. [42]	A 20-year-old male homozygous for the M694 V mutation affected by secondary amyloidosis (SAA) who developed signs of gastrointestinal toxicity to colchicine.	Anakinra allowed the control of both SAA and CRP levels. The patient remained clinically stable after several months.
Belkhir et al. [43]	A 68-year-old woman with FMF homozygous for the M694 V mutation of the MEFV gene was refractory to colchicine treatment and bedridden.	She responded clinically to Anakinra, and remained stable after 5 months.
Gattringer at al [45].	A 29-year-old woman was having recurrent FMF attacks despite being on colchicine for 26 years.	Administration of anakinra every second day improved the clinical and laboratory markers except when dose was interrupted.
Gattringer et al. [45]	A 32-year-old woman homozygous for the M694 V mutation of the MEFV gene was having recurrent FMF attacks every 2 weeks despite being on colchicine.	Improvement in clinical and laboratory markers for 1 month. Drug was stopped because of local reactions at the site of the injections and pneumonia episode.
Kuijk et al. [46]	A 14-year-old FMF patient with mutated MEFV gene was unresponsive to colchicine therapy.	Clinical and laboratory markers improvement for a period of 9 months.
Calligaris et al. [44]	A 15-year-old was having recurrent FMF attacks despite high dose colchicine.	Interrupting Anakinra lead to a relapse. Re-introduction lead to a good quality of life for 15 months.
Mitroulis et al. [47]	A 34-year-old male FMF patient, homozygous for pyrin M694 V mutation, had signs colchicine resistance after several years of successful treatment.	The clinical benefits of anakinra pulses were observed in the following six-month period.
Roldan et al. [48]	A 9-year-old child heterozygote for E148Q mutation in the MEFV gene with inefficacy to colchicine treatment.	A favorable clinical and biochemical responses have persisted over 6 months with no side effects.
Moser et al. [49]	A 43-year-old male developed colchicine resistance after 13 years of diagnosis.	The symptoms and inflammatory markers settled after dose was adjusted because of a kidney transplantation. A good clinical outcome in a 20 months period.
Petropoulo et al. [50]	A 22-year-old male patient who underwent bone marrow transplantation for idiopathic aplastic anemia from his FMF-affected brother acquired the M694 V mutation.	The patient had recurrent FMF flares until 110 days after transplantation despite colchicine. Anakinra was started. In a 4-month follow-up, neither FMF crisis nor any side effect of anakinra treatment has been observed.
Ozen et al. [51]	Five children and an adult patient (3 female, 3 male) had recurrent FMF attacks despite regular regular colchicine.	The patients were given Anakinra or Etanercept. Etanercept was considered ineffective. Anakinra reduced the number of attacks and lowered the levels of acute-phase reactants for a 4-year period.
Alpay et al. [52]	A 52-year-old patient with FMF, secondary amyloidosis, and renal transplant was resistant to colchicine treatment.	Clinical and biochemical markers improved significantly in a 1-month period. The efficacy of anakinra persisted in the following 2-month period.
Bilginer et al. [53]	An 8-year old girl who had typical attacks of FMF (M694 V/M694 V) developed features of Behçet’s disease despite colchicine.	Complete remission of both diseases was observed. After 18 months of anakinra, proteinuria reappeared.
Hennig et al. [54]	A 35-year old male with FMF associated with serum amyloid A amyloidosis and chronic obstructive pulmonary disease developed pneumonia as a consequence of an FMF bout 1 week after stopping Anakinra.	Colchicine was suspended. On 4 consecutive days, the patient was treated with anakinra leading to immediate improvement of clinical symptoms, inflammation signs, and radiological findings.
Stojanovic et al. [55]	Four cases of patients with FMF-associated amyloidosis homozygous for the M694 V mutation were colchicine resistant.	Complete remission of attacks with improvement of biochemical markers for a follow-up period of at last 6 months.
Estublier et al. [56]	A 39-year-old man with FMF homozygous for the M694I mutation in the MEFV gene developed myositis of the right quadriceps muscle. He had frequent and severe arthralgias, despite colchicine, then etanercept and adalimumab, impairing his quality of life.	Anakinra therapy lead to dramatic improvement of muscular and articular symptoms. Patient remained stable for 10 months after the new therapy.
Soriano et al. [57]	A 63-year-old FMF male patient homozygous for M694 V mutation, affected by IgA nephropathy and type 2 diabetes mellitus with impaired renal function, developed colchicine resistance.	The 1-year follow-up confirmed patient’s good clinical conditions, with complete disappearance of FMF flares and stabilization of renal function. Re-appearance of mild myalgia after reducing anakinra to sub-optimal dose.
Celebi et al. [58]	46-year-old woman with FMF homozygote for M694 V mutation developed colchicine resistance after 26 years of usage.	At the eighth month of follow-up after anakinra initiation, the patient remained asymptomatic, and had good quality of life.
Mercan et al. [59]	A 41-year-old female patient with FMF heterozygous for M694 V mutation presented with severe calf and thigh pain. Her MRI revealed remarkable muscle edema in her calf muscles which was consistent with protracted febrile myalgia syndrome.	Patient was started on anakinra after refusing prednisone. Her complaints completely resolved after two doses of anakinra. Her ESR and CRP returned to normal levels within a week. No recurrence was observed in her 3-month follow-up.
Mercan et al. [59]	A 44-year-old female with FMF homozygous for M694 V mutation presented with severe right thigh pain. On the basis of her symptoms and findings she was diagnosed with PFMS. Her attacks were considered colchicine resistant.	She showed more than 50% improvement even after the first dose of anakinra and complete recovery after the second dose.
Sevillano et al. [60]	A 67-year-old patient with FMF and biopsy-proven amyloidosis presented with nephrotic syndrome despite colchicine treatment.	Anakinra was started and a dramatic complete remission of nephrotic syndrome was observed in the following months. Patient remained clinically and biochemically stable 42 months after treatment.
Ben-Zvi et al. [61]	25 patients with colchicine-resistant FMF were enrolled, of whom 12 were randomized to receive anakinra and 13 to receive placebo.	The mean number of attacks per patient per month was lower in those receiving anakinra. 6 patients in the anakinra group, compared to none in the placebo group, had < 1 attack per month. Patients on anakinra had a better quality of life. There were no severe adverse events over a 20-month period follow-up.
Pecher et al. [62]	13 patients with colchicine resistant FMF were enrolled.	5 patients reached complete remission, whereas the other 8 patients had a partial remission to anakinra. 1 patient quit anakinra intermittently owing to a lack of compliance. No serious adverse events was observed, except for one respiratory infection which required antibiotic treatment.
Ilgen et al. [63]	A 27-year-old female with heterozygous M680I/M694 V MEFV gene mutation had colchicine resistance after 13 years of use.	After 4 months of treatment with anakinra, she was attack-free and IVF was planned. She had a successful pregnancy. The infant at 13 months of age had normal anticipated development and was breastfed from the time of birth without any health problem. The mother was still on anakinra with no attacks after delivery.
Venhoff et al. [64]	Data from 3 patients with a total of 4 pregnancies under anakinra therapy were analyzed. All patients had a molecular genetic mutation in the MEFV gene. Due to treatment failure with colchicine therapy, anakinra was initiated.	In 3 pregnancies, Anakinra was continued throughout the pregnancy, and in 1 was only used in the second trimester of pregnancy for uncontrolled disease activity. The fetal development was inconspicuous in all pregnancies All children showed an inconspicuous early childhood development without evidence of an existing disease.
Laskari et al. [65]	14 patients (7 men) with genetically confirmed FMF, with median disease duration of 14 years and active disease despite colchicine (*n* = 9) or both colchicine and anakinra (*n* = 5), received Canakinumab 150 mg subcutaneously (sc) every 4 (*n* = 7) or 6 (*n* = 2) or 8 weeks (*n* = 5) for a median of 18 months.	11 out of 14 patients (79%) achieved complete clinical remission while normalization of all laboratory parameters associated with inflammation occurred in 92% of patients. The remaining patients achieved partial responses. Canakinumab was well tolerated; one patient experienced an urinary tract infection and another one a viral gastroenteritis. Follow-up period was 12 months.
Babaoglu et al. [66]	A total of 78 patients were treated with IL-1 inhibitors in the cohort. Among these, 15 patients were identified who received on-demand anakinra.	On-demand anakinra prevented progression of prodromes to full-blown attacks which was demonstrated by decrease in the rate of attack/prodrome ratio.

##### Canakinumab

Canakinumab is the only FDA-approved cytokine blocker for the treatment of colchicine-resistant FMF in the United States [67]. The first report in the literature of the successful administration of Canakinumab in a patient with FMF and chronic arthritis after failing Anakinra, Etanercept and low dose prednisone, and Methotrexate was published in 2011 by Mitroulis et al. [68]. Table 2 summarizes all case reports and studies describing the efficacy of Canakinumab in the treatment of FMF.

**Table 2:** Studies and case reports that discussed the use of Canakinumab in FMF

Study	Description	Follow-up
Mitroulis et al. [68]	A 25-year-old woman with FMF, homozygous for the MEFV gene mutation, and with longstanding articular involvement affecting her hips and left knee. She had been under treatment with colchicine since the age of 5. At the age of 19, she experienced long-lasting arthritis of her right hip and was treated with anakinra which was discontinued due to severe injection site reactions. She was switched to treatment with etanercept and low dose prednisone which induced a destructive arthritis of the right hip and chronic arthritis of her left knee. Methotrexate was added to the existing treatment.	Long-term remission was not achieved. The patient only responded to canakinumab.
Hacihamdioglu et al. [69]	A 14-year-old girl homozygote for the M694 V mutation in the MEFV gene became colchicine resistant after 4 years of starting treatment.	The patient improved clinically and biochemically after canakinumab initiation until day 70 when she had a relapse. A second dose was administered again with full response.
Brik et al. [70]	7 participants received 3 subcutaneous injections of canakinumab. The primary outcome measure was the proportion of participants with ≥50% reduction in the frequency of FMF attacks during the treatment period versus the pretreatment period. Secondary outcome measures included acute-phase reactant levels, health-related quality of life, physician’s global assessment of FMF control, time to attack following the last canakinumab injection, and safety and tolerability of canakinumab.	6 participants met the primary outcome measure with a ≥ 50% reduction (range 76–100%) in the rate of FMF attacks. 18 of 34 attacks (53%) were rated as severe or very severe during the pretreatment phase, compared with 0 of 8 during the treatment phase. There were no serious adverse events.
Alpa et al. [71]	A 30-year old woman with FMF on colchicine for 6 years developed recurrent attacks. Increased colchicine dose was ineffective.	The effects of canakinumab were monitored over the following 6-month period. A significant improvement in the clinical symptoms was observed after the first administration. During treatment with canakinumab, the patient reported only three minor episodes of FMF.
Gul et al. [72]	Patients who had an attack despite maximal dose of colchicine in the preceding 3 months were eligible for the study. At the first attack, patients started to receive three canakinumab 150 mg subcutaneous injections at 4-week intervals, and were then followed for an additional 2 months.	13 patients were enrolled in the run-in period and 9 advanced to the treatment period. All 9 patients achieved a 50% or more reduction in attack frequency. C-reactive protein and serum amyloid A protein levels remained low throughout the treatment period. Only 5 patients had an attack during the 2-month follow-up.
Sozeri et al. [73]	A 14-year-old male with colchicine-resistant FMF and amyloidosis homozygous for the M694 V mutation in MEFV gene.	Because of his poor response to colchicine, severe growth retardation, and severe proteinuria due to amyloidosis, canakinumab was started. After 26 months of follow-up, his complaints, inflammatory parameters, and proteinuria were decreased. No side effects were noted.
Ozkan et al. [74]	This study was performed with 4 patients with colchicine-resistant FMF. None of the patients had proteinuria or amyloidosis.	First, anakinra was given to all the patients in addition to colchicine and showed a rapid and dramatic effect on attacks of FMF and inflammatory findings. There were no side effects, but daily anakinra injection therapy was changed to monthly injection of canakinumab because of easy implementation.
Yaziltas et al. [75]	This study retrospectively evaluated 11 pediatric colchicine-resistant FMF patients who were treated with canakinumab. Three of the patients had amyloidosis and two had uveitis.	In all, three patients had FMF-associated amyloidosis, nephrotic syndrome, and CKD, of which 2 had clinical improvement with canakinumab and 1 had partial response. Among the patients, only 1 case died1 year after canakinumab cessation due to sepsis. 9 of our patients continued to receive canakinumab treatment.
Jesenak et al. [76]	A 36-year-old man from central Europe presented with a very severe clinical FMF and intolerance to colchicine. The clinical effect of the application of anakinra was insufficient and accompanied with side effects and low tolerability.	Switching to canakinumab induced a rapid remission of the disease activity and inflammatory markers.
De Benedetti et al. [77]	63 colchicine-resistant FMF patients were tested for the effectiveness of canakinumab. Primary outcome was described as the resolution of the index flare by day 15 and no new episodes over 16 weeks of treatment.	The primary outcome was achieved by 61% of patients in the treatment group compared to 6% in the placebo arm. No opportunistic infections, tuberculosis, or death was observed but 3 serious infections were reported in two patients.
Trabulus et al. [78]	A total of 9 kidney transplant recipients who developed AA amyloidosis due to FMF were enrolled.	All of the patients had rapid or gradual disappearance of FMF attacks. 1 patient developed a reaction to injection while another showed symptoms of Cytomegalovirus pneumonia.

There has been some relatively large randomized studies that tested the efficacy of Canakinumab and/or Anakinra in treating FMF attacks. Meizner et al. treated 7 patients with recurrent FMF attacks with Anakinra or Cankinumab along with colchicine on board. The regimen was beneficial to all patients (complete remission in 6 patients, partial remission in 1 patient) [79].

A study by Cetin et al. included 20 patients in whom Colchicine was considered ineffective. Twelve patients received anakinra, and 8 patients were treated with canakinumab. Only 1 patient did not respond to the Anakinra. A significant decrease in proteinuria in the amyloidosis complicated FMF patients was observed [80]. Basaran et al. analyzed the MEFV gene in 8 patients having refractory FMF. They found homozygous mutations in 6 patients. All patients were successfully treated with anakinra and/or canakinumab [81]. Fourteen patients were included in Eroglu et al. study, 11 of which were treated with anakinra. Nine patients responded to the treatment at the third month, but 4 of them switched to canakinumab because of noncompliance, local side effects, and active arthritis. Nine patients in total were treated with canakinumab. All patients treated with canakinumab responded well [82]. Thirteen patients were included in the study by Ozcakar et al. 7 of them received anti-IL-1 therapy due to colchicine resistance and 6 due to FMF-related amyloidosis. In all treated patients, attacks completely disappeared or decreased in frequency [83]. Anakinra and canakinumab showed a rapid (2 ± 3 days) and persistent suppression of FMF symptoms and inflammatory parameters in 31 colchicine resistant FMF patients. The frequency of FMF attacks was significantly reduced [84]. Kucuksahin et al. followed up patients using colchicine for 4 months to 30 years. In some patients, the treatment was switched to anti-IL-1 treatment for various reasons. Twenty-four patients used anakinra and 2 used canakinumab. Sixteen patients with colchicine resistance had no attacks under anti-IL-1 treatment, and 4 had decreased frequency and duration of attacks [85]. Varan et al. treated 33 patients with anakinra and 11 with canakinumab. Striking improvements were detected in frequency, duration, and visual analog scale (VAS) severity of attacks [86]. Also, Varan et al. identified 17 patients with colchicine resistant FMF-amyloidosis. Background colchicine therapy was continued in all patients in maximal-tolerated dose along with IL-1 inhibitors. All patients benefited from IL-1 antagonists assessed by patient and physician global assessments. Inflammatory markers and the amount of proteinuria were reduced in all patients [87].

##### Rilonacept

In February 2008, rilonacept received the approval from the FDA for the treatment of two Cryopyrin-associated periodic syndrome (CAPS) disorders, namely, familial cold-induced autoinflammatory syndrome (FCAS) and Muckle-Wells syndrome (MWS), for children and adults 12 years and older [88]. As a primary study to assess the efficacy and safety of rilonacept in treating patients with colchicine-resistant FMF, Hashkes et al. [89] performed a randomized, double blind, placebo-controlled trial including 14 patients. The complete remission was observed in two patients during the 3-month treatment course, while eight patients had a partial response. The remaining four had no significant reduction in attack frequency [89]. There was no reported serious side effect for rilonacept in this study. Table 3 lists the 3 studies that discussed the successful treatment of FMF with rilonacept.

**Table 3:** Studies and case reports that discussed the use of Rilonacept in FMF

Study	Description	Follow-up
Hashkes et al. [89]	8 males and 6 females were randomly assigned to one of 4 treatment sequences that each included two 3-month courses of rilonacept by weekly subcutaneous injection, and two 3-month courses of placebo.	During the treatment periods with rilonacept, patients had fewer attacks of FMF than with placebo, and more patients had a decrease to less than 50% of their previous baseline number. Patients also reported better physical aspects of quality of life with rilonacept. Patients had pain with the rilonacept injections more often than with placebo.
Hashkes et al. [90]	14 FMF patients with their parents completed the modified Child Health Questionnaire at baseline, and at the start and end of each of 4 treatment courses, 2 each with rilonacept and placebo.	There were significant improvements in most health-related quality of life concepts after rilonacept but not placebo. Significant differences between rilonacept and placebo were found in the physical but not psychosocial scores.
Sakallioglu et al. [91]	A case of a 15 year-old boy with polyarthritis while on colchicine treatment for FMF. His polyarthritis was resistant to treatment with prednisolone and methotrexate when etanercept was started.	He responded dramatically to etanercept and remained in full remission until 4 months when the drug was discontinued due to social and financial causes.

#### Anti TNF drugs

In 1991, Schattner et al. [92] studied the levels of tumor necrosis factor (TNF) in the plasma and in supernatants of peripheral blood mononuclear cells (PBMC) incubated alone or with an inducer in 36 asymptomatic and 24 patients with acute FMF and compared with 20 matched healthy subjects. No TNF was found in plasma and non-induced PBMC supernatants. Induced TNF production was markedly decreased in patients with acute FMF and increased in asymptomatic FMF patients to levels over those of control subjects. Re-testing of patients first studied during an acute episode when their disease was quiescent revealed a fivefold increase in TNF production. The capacity of PBMC to respond to TNF inducers may more accurately reflect its synthesis. The marked decrease in the response of PBMC to TNF inducers in acute FMF suggested that cells were already exhausted and highly activated to produce TNF possibly contributing to the pathogenesis of FMF. Later on, other quantitative studies have been published on the role of TNF-α in FMF. Those studies reported a decreased/slightly increased TNF-α levels during acute attacks or normal/increased levels between the attacks [93–96]. Gang et al. [97] found increased levels of soluble TNF receptor fusion protein p55 and p75 during attacks. Then, it was found out that the MEFV gene is upregulated by TNF-α [98]. Lachman et al. reported the first case, where a 38-year-old FMF patient with protracted arthritis responded favorably to infliximab. Sakallioglu et al. [91] presented a case of successful use of etanercept on a pediatric FMF patient resistant to colchicine, steroid, and methotrexate. Another report by Ozgocmen et al. [99] described the successful use of adalimumab in 3 patients with FMF. Table 4 summarizes all case reports and studies describing the use of anti-TNF drugs in treating the manifestations of FMF.

**Table 4:** Studies and case reports that discussed the use of Anti-TNF drugs in FMF

Study	Description	Follow-up
Aldea et al. [100]	3 patients with fever, arthritis, abdominal attacks, and rash along with amyloidosis for 1 patient were treated with infliximab.	The attacks were discontinued or significantly improved. Patients were followed-up for 12 months except for the amyloidosis patient for 3 months.
Metyas et al. [101]	A 30-year-old presented with recurrent fever, weight loss, intermittent bilateral knee pain and swelling, abdominal pain with intermittent vomiting, and chest pain. Amyloidosis was confirmed by Congo red stain and immunohistochemical stains. The patient was refractory to methotrexate treatment.	By the sixth dose of infliximab, the patient felt dramatically better. Arthritis, swelling, chest pain, and abdominal pain resolved. The patient was able to return to work after 2 years of disability secondary to arthritis. Laboratory markers of amyloidosis improved.
Kaya et al. [102]	A 27-year-old male patient with FMF and Juvenile Idiopathic Arthritis (JIA) heterozygote for M694 V mutation presented for a relapse of his rheumatological diseases. He was started on menthotrexate and colchicine with steroid on board. Leflunomide was added later due to partial response to methotrexate.	At the 7th month of MTX and leflunomide treatment, Etanercept was started as joint inflammation did not subside despite methotrexate and leflunomide. His FMF attacks were improved; he had only one attack of abdominal pain after the onset of etanercept treatment. Follow-up period was 2 months.
Ozgocmen et al. [103]	A 35-year-old woman with FMF resistant to colchicine failed a combination of colchicine, sulfasalazine, and methotrexate. Infliximab was added to the treatment regimen.	A 72-week follow-up of the patient yielded complete remission of the febrile abdominal episodes and spondylitis. The patient’s bilateral aseptic necrosis of the femoral head deteriorated and caused hip pain, discomfort, and disability.
Daysal et al. [104]	A 21-year-old female homozygote for M694 V mutation presented for severe hip arthritis and elevated inflammatory markers which didn’t improve with colchicine, steroid, hydroxychloroquine and methotrexate.	Infliximab was given intravenously. She had a prompt response. In 4–5 days, she was able to ambulate independently. Two weeks later her ESR and CRP were negative, and steroid therapy was discontinued. Follow-up period was 2 months.
Mor et al. [105]	A 35-year-old man presented for episodes of articular pain, episodic testicular pain, recurrent oral ulcers, episodic abdominal pain, and episodic fever. He had elevated inflammatory markers. A single V726A mutation in the MEFV gene was identified.	Colchicine was discontinued because of diarrhea. The patient was started on etanercept 25 mg twice a week, which immediately reduced the frequency, duration, and severity of the joint attacks for 3 years.
Nakamura et al. [106]	A Japanese patient with FMF has a heterozygous mutations in the MEFV gene. Conventional treatment, such as colchicine and reserpine, failed to sufficiently control the FMF attacks.	After starting infliximab and low-dose methotrexate, the frequency of the FMF attacks dramatically decreased and the clinical effect has remained unchanged for longer than 1 year.
Ozgocmen et al. [99]	The patients were a 37-year-old man with concomitant Crohn’s disease and amyloidosis who was treated with infliximab and switched to adalimumab, and two female patients (a 24-year-old and a 31-year-old) with FMF who developed severe spondylitis.	Adalimumab controlled FMF attacks quit effectively.
Bilgen et al. [107]	The data for 10 FMF patients (5 male and 5 female patients) with chronic arthritis and/or sacroiliitis who were on anti-TNF agents. Frequency of FMF attacks before and after treatment with anti-TNF agents was recorded. The effects of the anti-TNF treatment were determined by using the number of tender and/or swollen joints, serum acute phase reactant levels, and Bath Ankylosing Spondylitis Disease Activity Index scores. Change in urine protein loss was also evaluated in patients with amyloidosis.	After anti-TNF treatment, in 3 patients, FMF attack frequency decreased, and in the remaining 7 patients, no attack occurred. Serum acute phase reactant levels were decreased significantly at 3 and 6 months after anti-TNF treatment. After anti-TNF treatment Bath Ankylosing Spondylitis Disease Activity Index scores were also decreased significantly. In all 3 patients with amyloidosis, urine protein loss decreased without any increase in serum creatinine levels.
Erten et al. [108]	A 35-year-old male patient presented with fever, abdominal pain, malaise, and low back and ankle pain. He was on colchicine and sulfasalazine for FMF with homozygous M694 V mutation. Infliximab treatment was discontinued due to allergic symptoms.	The patient responded well to etanercept. Febrile abdominal attacks and joint symptoms did not recur. Urinary proteinuria and acute phase proteins returned to normal limits. He remained in an excellent condition for 4 years of follow-up.
Kosmidou et al. [109]	A 33-year-old male patient heterozygote for M694 V mutation had been diagnosed with FMF since his adolescence. He presented for atypical abdominal pain to be diagnosed with Crohn’s disease. Steroid and azathioprine were started. 2 months after the initiation of immunosuppressive treatment, there was still no favorable response on his bowel inflammation.	In a 6-month extensive follow-up after starting infliximab, both diseases were found to be inactive.

#### Anti IL6 drugs

In clinical settings, tocilizumab (TCZ), an IL-6 receptor blocker, has been widely used for the treatment of rheumatoid arthritis (RA). The first cases reported on the success of tocilizumab in the treatment of FMF came from Japan [110–112]. Yilmaz et al. [113] reported 11 cases with AA amyloidosis secondary to FMF successfully treated by TCZ. Among these 11 patients, 10 patients did not experience any attack during the course of treatment, and no major adverse events were observed. Even though 8 patients had the decreased level of the proteinuria after treatment, there was no case where the deposition of amyloid in any organ have been confirmed to be reduced by biopsy. Table 5 summarizes all the case reports and studies which discuss the successful treatment of FMF manifestations with TCZ.

**Table 5:** Studies and case reports that discussed the use of toclizumab in FMF

Study	Description	Follow-up
Fujikawa et al. [110]	A 19-year-old female presented with fever, a sore throat, polyarthralgia, and fever-associated skin rashes lasting a couple of days. Inflammatory markers were elevated. Patient was heterozygote for MEFV gene mutation. Steroid and methotrexate didn’t improve the patient’s symptoms.	TCZ treatment was combined with the steroid and methotrexate therapy. The patient’s symptoms, including skin rash, disappeared promptly, so steroid and methotrexate treatment were tapered and then stopped. TCZ was then stopped. Patient remained symptom free for 4 months when he had a relapse managed by colchicine.
Yilmaz et al. [113]	11 patients who had AA amyloidosis secondary to FMF with renal involvement and were treated with TCZ. Before TCZ therapy, all the patients had been using colchicine and an angiotensin receptor blocker or angiotensin converting enzyme inhibitor for at least 3 months without significant response.	Among the 11 patients, 10 patients did not experience any attack during the course of treatment, and no major adverse events were observed. Even though 8 patients had the decreased level of the proteinuria after treatment, there was no case confirmed the reduction of deposition of amyloid in any organ.
Hamanoue et al. [111]	A 51-year-old Japanese man with suspected FMF had periodic fever with abdominal pain, polyarthritis, and nephropathy. FMF was diagnosed by mutation analysis of the MEFV gene. AA amyloidosis was diagnosed by renal and gastric biopsy. Colchicine was administered, but his arthritis persisted, and serum creatinine increased.	Tocilizumab was administered on a monthly basis. Both arthritis and abdominal pain subsided rapidly, and CRP decreased. After 2 years, his serum creatinine was decreased and proteinuria was improved. Repeat gastric biopsy showed a marked decrease of AA amyloidosis.
Umeda et al. [112]	A 64-year-old Japanese woman had been treated with prednisolone for 7 years for undiagnosed recurrent fever and myalgia. She had elevated liver enzymes and inflammatory markers. A muscle biopsy of left quadriceps revealed the infiltration of neutrophils into the fascia as well as vascular endothelium of fascia. She was hetrozygote for the MEFV gene mutation. Patient was stable on colchicine for 5 months when she had a relapse.	Since the initiation of this TCZ treatment, the FMF attacks of fever and myalgia have been completely inhibited for 9 months.
Nikiphorou et al. [114]	A 20-year-old man with a history of chronic recurrent multifocal osteomyelitis, aseptic multiphasic disseminating encephalomyelitis, and a single mutation in the MEFV gene. Despite corticosteroids, adalimumab, colchicine, mycophenolate, and hydroxyxhloroquine normocytic anaemia and high acute phase response persisted.	A trial of anakinra resulted in only partial clinical response, leading to tocilizumab as the next treatment choice. This resulted in rapid clinical and biochemical improvement. After 5 years with ongoing tocilizumab and colchicine he remained well, with normal CRP and amyloid levels.
Nikiphorou et al. [114]	An 18-year-old man had multiple admissions for meningoencephalitis, pyrexia, and anemia. Genetic testing revealed heterozygosity for the MEFV gene.	Sirolimus was unsuccessful in controlling symptoms, leading to a trial of tocilizumab which resulted in suppression of CRP and amyloid to normal levels and enabled steroid withdrawal.
Aikawa et al. [115]	A 53-year-old man with recurrent episodes of large joint pain and a low-grade fever at irregular intervals for 16 years developed right knee and ankle arthralgia, watery diarrhea, and abdominal pain. Following an ileum and colon biopsy, he was diagnosed with gastrointestinal amyloidosis. FMF was suspected, and colchicine was administered colchicine; his symptoms subsequently improved. The patient had atypical FMF. Colchicine was stopped because of alopecia.	Remission was maintained by tocilizumab therapy. In addition, the amyloid deposition in the ileum and colon disappeared.

#### Januse kinase inhibitors

Januse kinase inhibitors have been well studied for the treatment of RA [116]. Tofacitinib (Xeljanz) is specific to the JAK-STAT pathway with preferential inhibition of JAK1 and JAK3 [117]. Recently, Gok et al. [118] described recently the case of a 27-year-old woman with RA and FMF resistant to colchicine who presented for morning stiffness. She had elevated inflammatory markers, and was started on sulfasalazine, hydroxychloroquine, methotrexate, and steroid. After 3 months of the regimen, the patient continued to have the attacks. The patient was followed for 12-months under treatment with tofacitinib and colchicine. She was completely attack free, and no adverse events occurred. This case report is promising for the use of janus kinase inhibitors to control colchicine-resistant FMF attacks.

## Conclusions

Familial Mediterranean Fever (FMF) is the most common autoinflammatory disease. A mutation of the MEFV gene on chromosome 16, which codes for protein pyrin, is associated with the disease pathogenesis. Colchicine, which has been prescribed to treat FMF since 1972, remains the mainstay for treatment although its use has been complicated by resistance and intolerance in a minority of patients. Since FMF patients have high levels of certain cytokines, practitioners have found in biologics a solution for colchicine resistant and intolerant cases given the success that biologics have shown in other autoimmune and auto-inflammatory diseases. Anti-interleukin 1, anti-interleukin 6, anti-TNF, and Janus Kinase inhibitors drugs, can be beneficial add-on to colchicine in treating FMF manifestations.

## Data Availability

Data sharing is not applicable to this article as no datasets were generated or analysed during the current study.

## Abbreviations

AID: Autoinflammatory disease
CAPS: Cryopyrin-associated periodic syndrome
EULAR: European League Against Rheumatism
FCAS: Familial cold-induced autoinflammatory syndrome
FMF: Familial Mediterranean fever
JIA: Juvenile Idiopathic Arthritis
MWS: Muckle-Wells syndrome
PBMC: Peripheral blood mononuclear cells
RA: Rheumatoid arthritis
SAA: Secondary amyloidosis
SAA: Serum Amyloid A
sc: Subcutaneously
SLE: Systemic lupus erythematosus
TCZ: Tocilizumab
TNF: Tumor necrosis factor
VAS: Visual analog scale
